# Supplementary material for: Splice-Junction-Based Mapping of Alternative Isoforms in the Human Proteome
Source: Cell Rep. Author manuscript; Available in PMC 2020 Jan 15. (PMC6961840; doi:10.1016/j.celrep.2019.11.026)
Supplement: 2 [file NIHMS1546469-supplement-2.zip › DF1/PXD010154/Prostate/Prostate_2_CLTCL1_LQEHFQLQNLGINPANIGFSTLTMESDK.pdf]

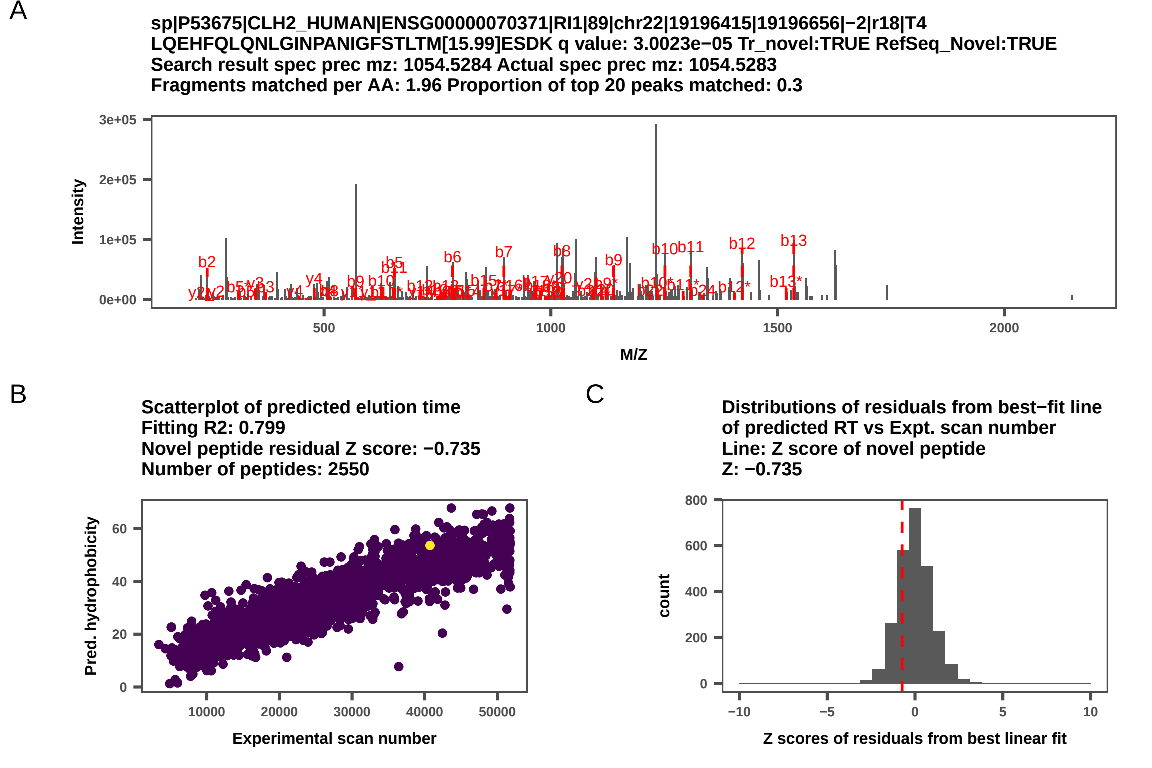
A
sp|P53675|CLH2_HUMAN|ENSG00000070371|RI1|89|chr22|19196415|19196656|−2|r18|T4
LQEHFQLQNLGINPANIGFSTLTM[15.99]ESDK q value: 3.0023e−05 Tr_novel:TRUE RefSeq_Novel:TRUE
Search result spec prec mz: 1054.5284 Actual spec prec mz: 1054.5283
Fragments matched per AA: 1.96 Proportion of top 20 peaks matched: 0.3
Intensity
3e+05
2e+05
1e+05
0e+00
b2
b6
b7
b8
b9
b10
b11
b12
b13
b13*
b12*
500
1000
1500
2000
M/Z
B
Scatterplot of predicted elution time
Fitting R2: 0.799
Novel peptide residual Z score: −0.735
Number of peptides: 2550
Pred. hydrophobicity
60
40
20
0
10000
20000
30000
40000
50000
Experimental scan number
C
Distributions of residuals from best−fit line
of predicted RT vs Expt. scan number
Line: Z score of novel peptide
Z: −0.735
count
800
600
400
200
0
−10
−5
0
5
10
Z scores of residuals from best linear fit
